# Supplementary material for: Determinants of receptor tyrosine phosphatase homophilic adhesion: Structural comparison of PTPRK and PTPRM extracellular domains
Source: J Biol Chem. 2022 Nov 25;299(1):102750. doi: 10.1016/j.jbc.2022.102750 (PMC9800333; doi:10.1016/j.jbc.2022.102750)
Supplement: Supporting infromation [file mmc1.pdf]

Supporting Information

Determinants of receptor tyrosine phosphatase homophilic adhesion: structural comparison of PTPRK and PTPRM extracellular domains

Iain M. Hay[1,2,3], Maria Shamin[1], Eve R. Caroe[1,4], Ahmed S. A. Mohammed[5], Dmitri I. Svergun[5], Cy M. Jeffries[5], Stephen C. Graham[6], Hayley J. Sharpe[2]* and Janet E. Deane[1]*

[1] Cambridge Institute for Medical Research, University of Cambridge, Cambridge, CB2 0XY, UK.

[2] Signalling Programme, Babraham Institute, Babraham Research Campus, Cambridge CB22 3AT, U.K.

[3] Current address: MRC Laboratory of Molecular Biology, Cambridge CB2 0QH, U.K.

[4] Current address: Retroviral Replication Laboratory, Francis Crick Institute, London NW1 1AT, U.K.

[5] European Molecular Biology Laboratory (EMBL) Hamburg Site, Hamburg, Germany.

[6] Department of Pathology, University of Cambridge, Tennis Court Road, Cambridge CB2 1QP, UK.

* Corresponding authors: Hayley Sharpe (hayley.sharpe@babraham.ac.uk) and Janet Deane (jed55@cam.ac.uk)

This Supporting Information file contains: Supplemental Figures 1 to 5, Supplemental Tables 1 and 2 and Supplemental References.

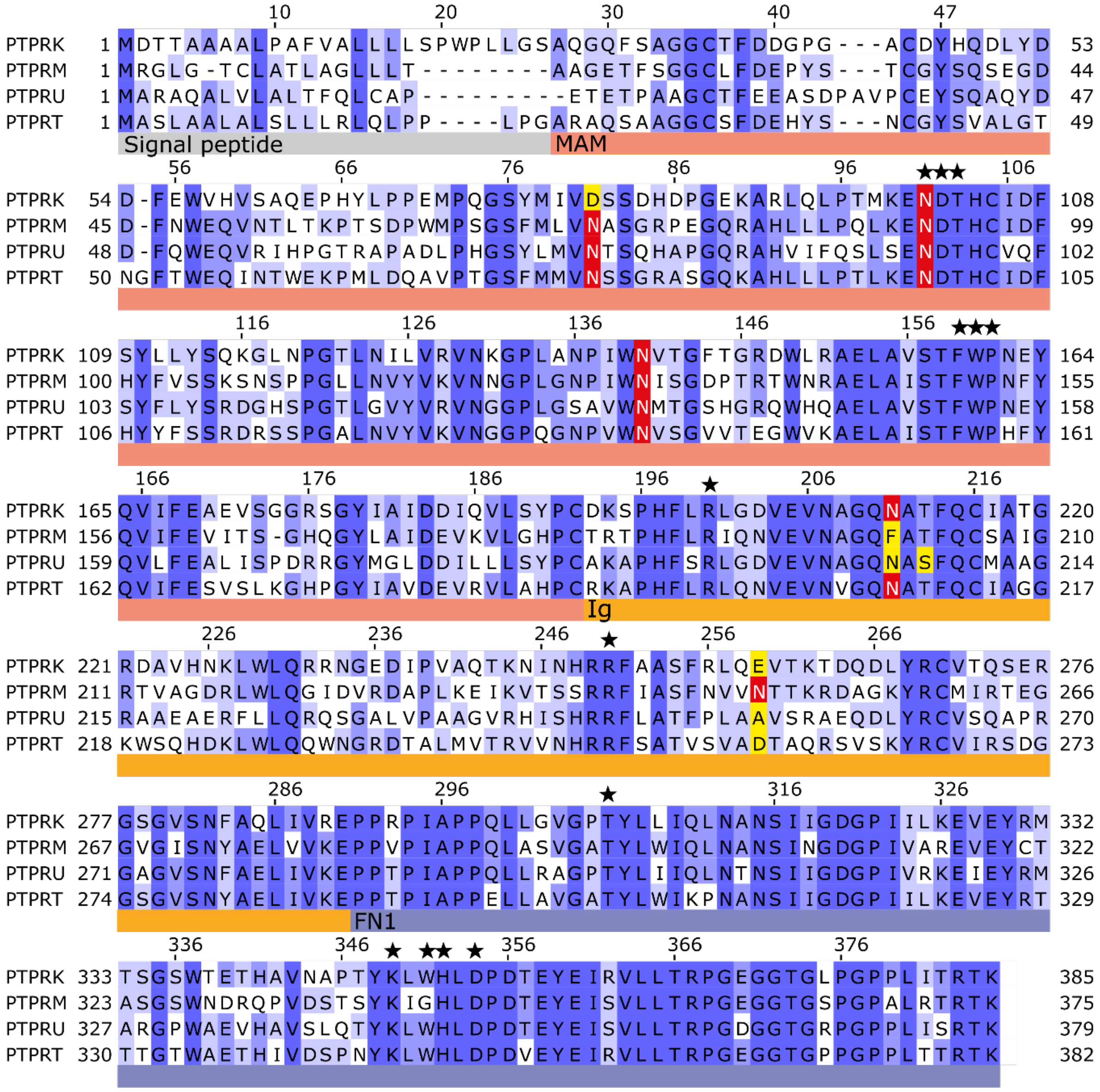

Fig. S1. Sequence alignment of the MAM, Ig and FN1 domains of the ECDs from the four R2B PTP family members. Multiple sequence alignment of the MAM (pink), Ig-like (Ig, orange) and first fibronectin type-III (FN1, blue) domains of the human R2B RPTPs, coloured by percentage identity (white-blue, 0-100% identity). Predicted N-glycosylation sites were identified using the NetNGlyC server (49) and are highlighted in red, with non-conserved sites in yellow. Residues involved in key intermolecular interactions at the MIFN1 dimer interface are highlighted (stars).

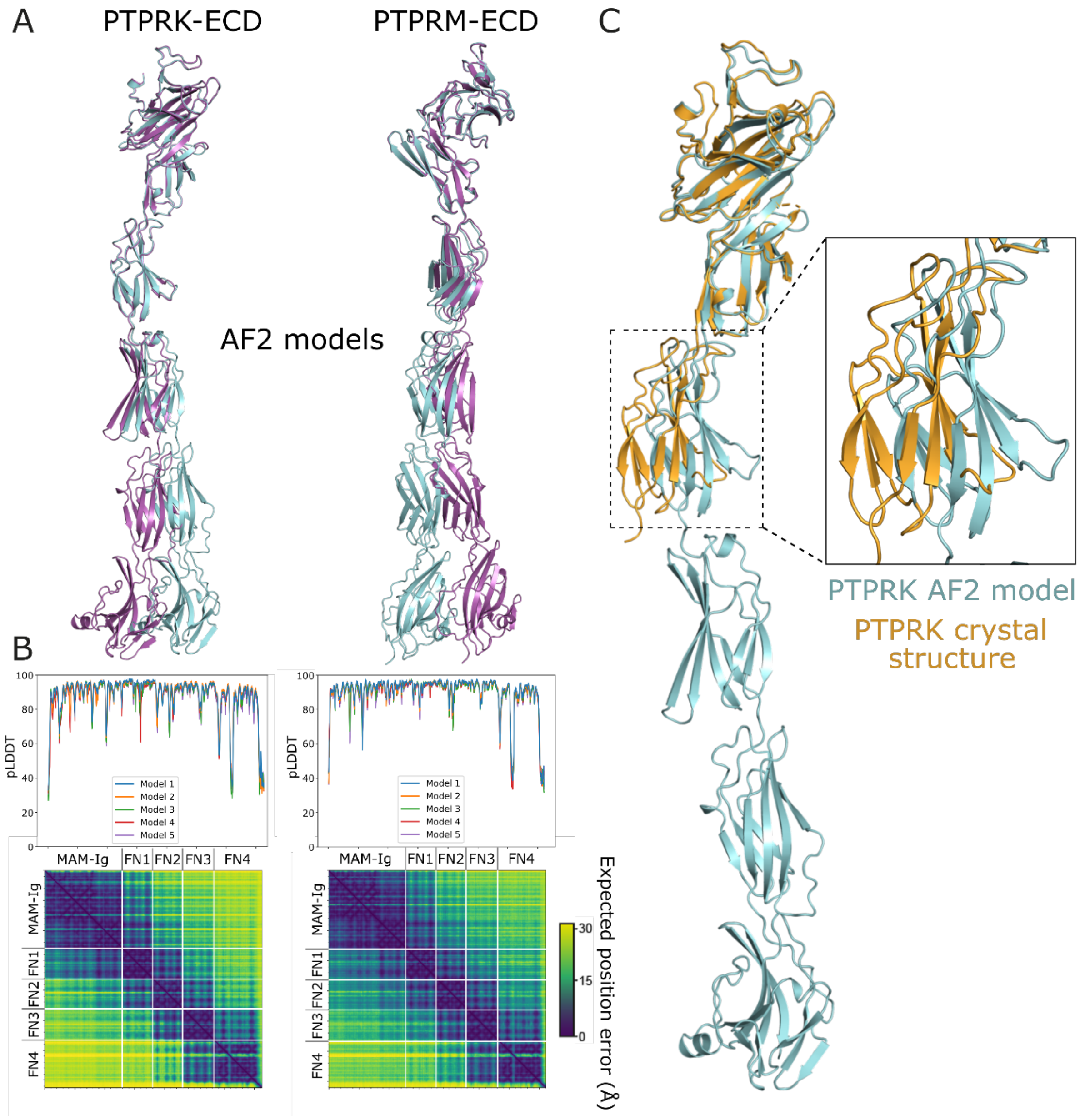

Fig. S2. AlphaFold2 (AF2) models of the PTPRK and PTPRM ECDs. A. For the PTPRK-ECD (left) and PTPRM-ECD (right) the top five AF2 models were superposed using the MAM-Ig domains as the reference for alignment. Two of the five models are displayed for clarity to demonstrate the range of long-distance conformations present in the ensemble of predicted structures (cyan and magenta). **B.** *Top,* pLDDT plots demonstrating good per-residue confidence for all PTPRK and PTPRM ECD models. *Bottom*, Representative Predicted Aligned Error (PAE) plots for PTPRK and PTPRM, with domain boundaries marked (white lines), demonstrating the low confidence (high error, yellow) of long-distance predictions. **C.** Alignment of the AF2 model for the PTPRK-ECD with the crystal structure of the PTPRK-MIFN1 using the MAM-Ig domains as the reference for alignment.

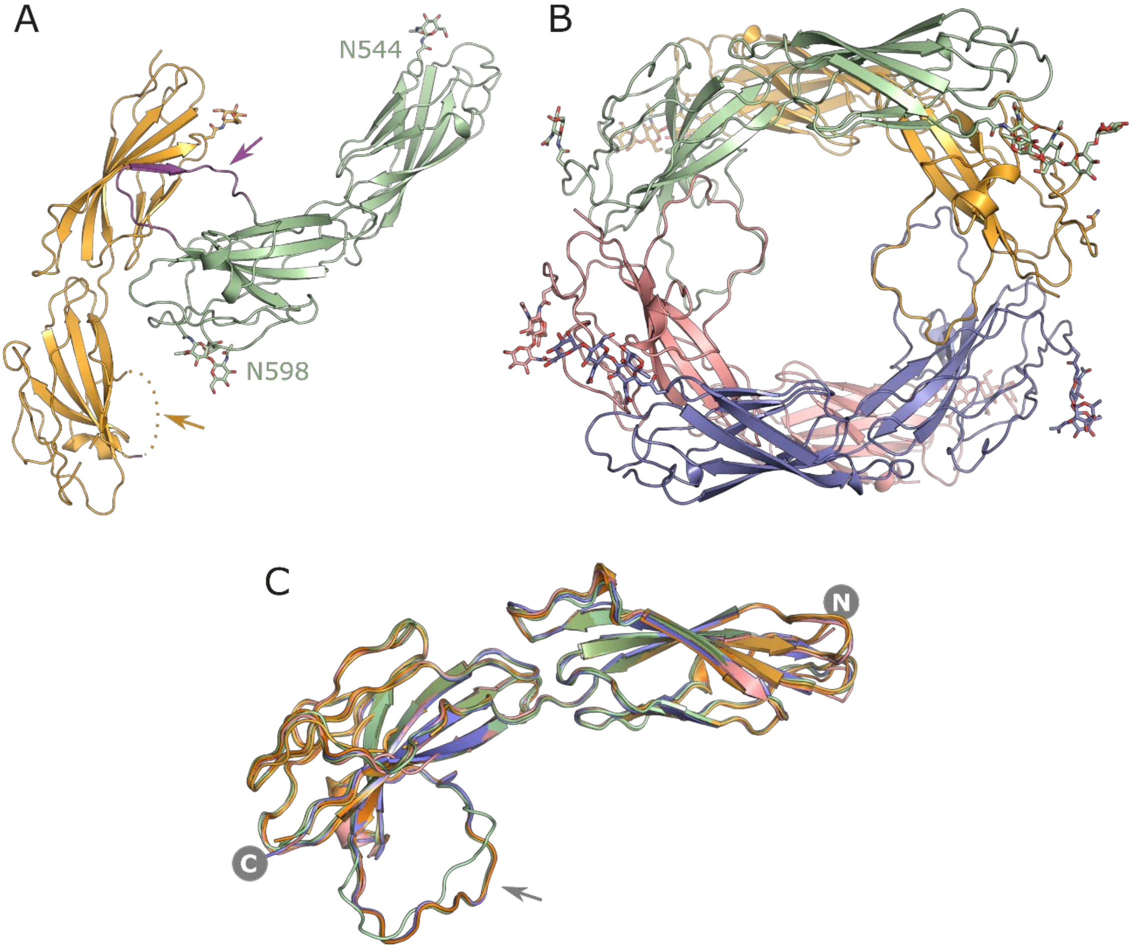

Fig. S3. Tertiary assemblies of the PTPRM FN3-4 structures determined in two different spacegroups. A. In $P2_12_12_1$ two molecules were present in the asymmetric unit (ASU). In one chain (orange) a loop encompassing residues 628–641 could not be modelled (dotted line) while in the second chain (green) this loop (purple, arrow) was ordered via interactions with the other chain. N-linked glycans are displayed as sticks on residues N544 in both chains and on N598 in the second chain only. **B.** In the $P3_221$ crystal form, four molecules of FN3-4 were arranged in a tetrameric assembly such that the loop (residues 628–641, arrows) in each chain was ordered via interactions with an adjacent chain. N-linked glycans are displayed as sticks on residues N544 and N598. **C.** Superposition of all six chains from the two crystal structures of PTPRM FN3-4. The furin cleavage site is indicated (gray arrow).

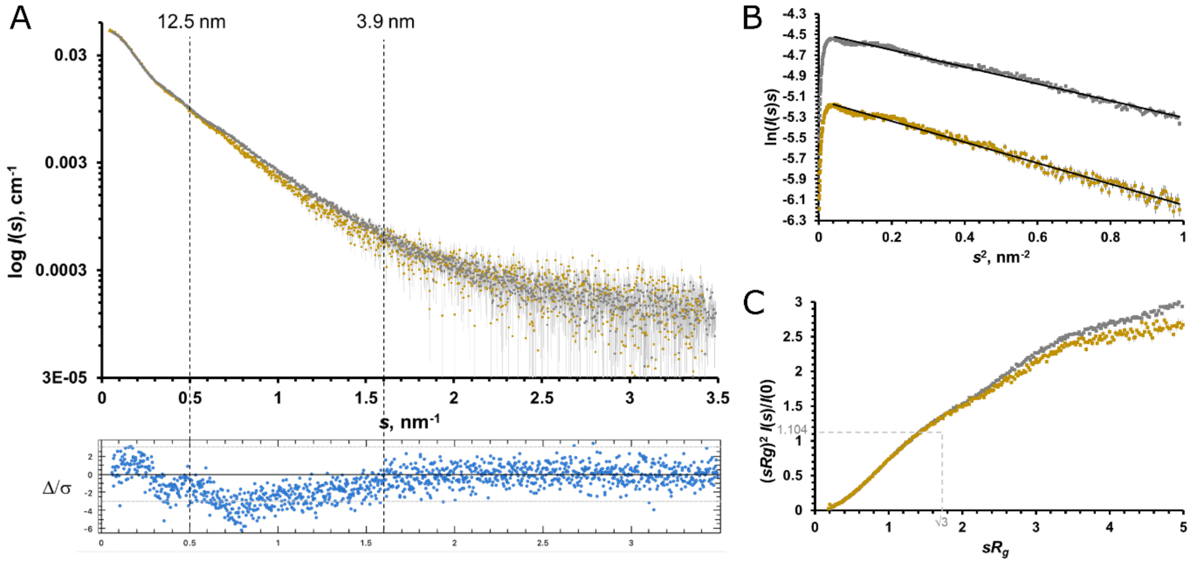

Fig. S4. Comparison of PTPRM-ECD and PTPRK-ECD SAXS profiles. A. Scaled SAXS data profiles measured from monomeric PTPRK-ECDs (orange) and PTPRM-ECDs (gray) showing differences in the scattering intensities at lower scattering angles, and especially between $0.5 < s < 1.6$ nm^{-1} corresponding to real-space distances spanning 12.5–3.9 nm. The error-weighted residual difference plot is displayed underneath, demonstrating systematic deviations between the two datasets for $s < 1.6$ nm^{-1}. **B.** Modified Guinier plots used to calculate the R_g^c for PTPRK-ECDs (orange) and PTPRM-ECDs (gray). The slope of the linear correlation (black line) is slightly steeper for the PTPRK-ECDs, that have a slightly larger R_g^c (1.34–1.39 nm) compared to the PTPRM-ECDs (1.2–1.25 nm). **C.** Dimensionless Kratky plots calculated for PTPRK-ECDs (orange) and PTPRM-ECDs (gray) showing a monotonic increase that is typical of extended structures (unlike compact or semi-flexible modular proteins that often have a maxima in the plot near √3, 1.104).

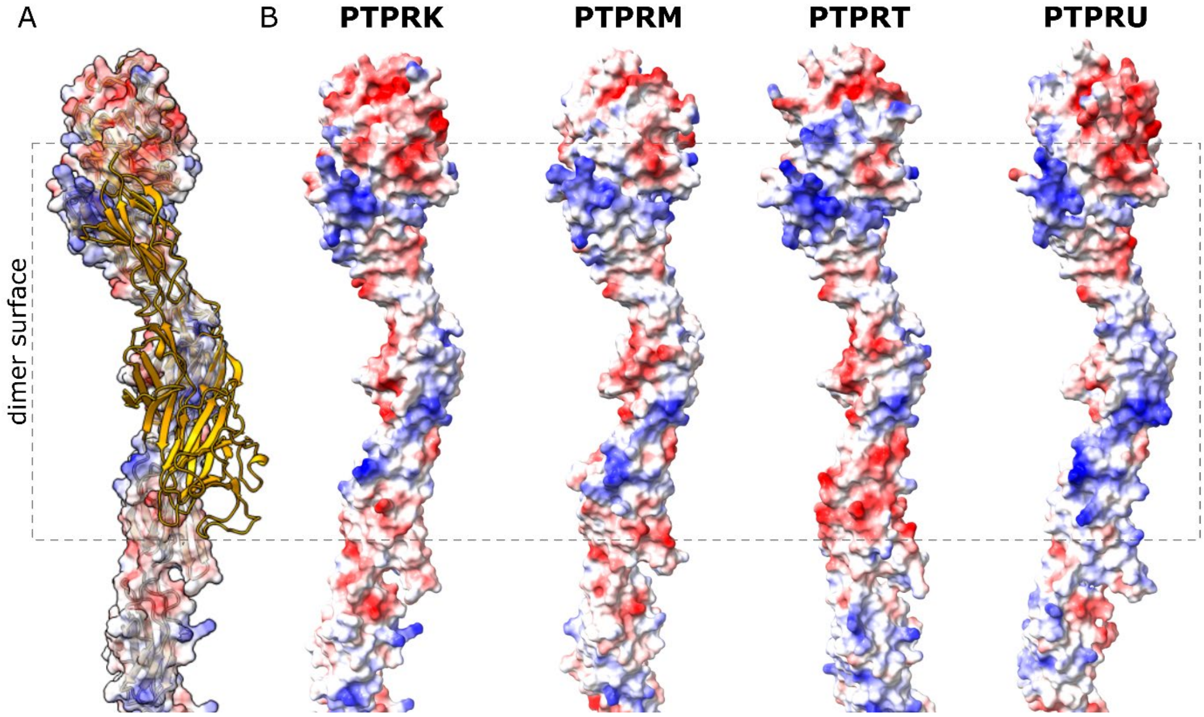

Fig. S5. Electrostatic surface charge at the interaction interface of models for all R2B family members. A. Surface representation of PTPRK ECD with a ribbon diagram of the second chain in the PTPRK-MAMIgFN1 dimer structure (orange) illustrating the orientation of the dimer interface. **B.** Surface electrostatics of AF2 models for the ECDs of PTPRK, PTPRM, PTPRT and PTPRU. The orientation allows direct comparison of the surface involved in dimer formation (grey dashed box).

Table S1. X-ray diffraction data collection and structure refinement. Data in parentheses relate to the highest resolution shell.

	**PTPRK-MIFN1**	**PTPRM-FN3-4**	**PTPRM-FN3-4**
**Data collection**			
Beamline	I04	I04-1	I03
Wavelength (Å)	0.97951	0.9159	0.9763
Space group	$P\,2_1\,2_1\,2_1$	$P\,2_1\,2_1\,2_1$	$P\,3_2\,2\,1$
Cell dimensions			
a,*b*,*c* (Å)	87.79, 93.86, 181.74	50.68, 89.64, 128.29	87.63, 87.63, 311.57
α,β,γ (°)	90, 90, 90	90, 90, 90	90, 90, 120
Resolution (Å)	79.05–3.00 (3.05–3.00)*	128.29–2.89 (2.94–2.89)	77.89–3.08 (3.14–3.08)
R_{merge}	0.079 (2.023)	0.317 (3.531)	0.170 (2.322)
R_{pim}	0.023 (0.577)	0.093 (0.996)	0.057 (0.819)
$CC_{1/2}$	1.000 (0.561)	0.991 (0.432)	0.993 (0.391)
$I/\sigma I$	18.7 (1.3)	7.0 (0.9)	9.1 (0.4)
Completeness (%)	100 (99.1)	100 (96.7)	99.8 (92.0)
Multiplicity	13.3 (13.1)	12.4 (13.3)	10.0 (8.5)
**Refinement**			
Resolution (Å)	60.46–3.00 (3.09–3.00)	73.48–2.89 (3.11–2.89)	75.80–3.09 (3.20–3.09)
No. reflections	30809	13636	26111
R_{work}/R_{free}	0.228/0.274	0.234/0.288	0.243/0.290
Molecules per ASU	2	2	4
No. atoms			
Protein	5543	3684	7692
Glycans	183	125	297
Other	4	0	0
B-factors			
Protein	116.0	75.1	112.6
Glycan	145.7	94.9	135.8
Other	120.65	0	0
Ramachandran			
Favoured (%)	98.0	97.8	97.9
Outliers (%)	0.0	0.0	0.0
r.m.s. deviations			
Bond lengths (Å)	0.003	0.003	0.012
Bond angles (°)	0.735	0.779	1.360
PDB entry	8A1F	8A16	8A17

Table S2. SAXS data collection and analysis parameters.

**Sample details**	**PTPRK**	**PTPRM**
Organism	*Human*	*Human*
Uniprot ID (amino acid range)	Q15262 (28–752)*	P28827 (19–742)
SEC-SAXS buffer	50 mM MES, pH 6.0, 250 mM NaCl, 3% v/v glycerol	
Sample injection volume	40 µL	30 µL
Sample injection conc.	2.3 mg/mL	7 mg/mL
SEC column	S200 Increase 5/150	
SEC flow rate	0.35 mL/min	
SEC temperature	20ºC	
**Instrument details**		
Instrument	EMBL P12 bioSAXS beam line, DESY, Hamburg	
Exposure time (# frames)	0.25 s (2880), entire column elution	
X-ray wavelength/energy	0.124 nm (10 keV)	
Sample-to-detector distance	3 m	
Scattering intensity scale	*Absolute scale, cm^{-1}*	
SEC-SAXS primary data processing	*CHROMIXS*	
# frames used for averaging	47	54
Working *s*-range (nm^{-1})	0.04–7.4	0.06–7.4
**Guinier analysis**		
Primary data analysis software	*PRIMUS* (*ATSAS* 3.0)	
Guinier $I(0)$ (σ)	0.0523 (0.0001)	0.1013 (0.0002)
R_g, Guinier, nm (σ)	7.0 (0.02)	7.2 (0.02)
sR_g range (points used)	0.29–1.28 (7–58)	0.23–1.23 (4–54)
R_g^c, cross-section, nm	1.34–1.39	1.20–1.25
***p*(*r*) analysis**		
Method	*GNOM* 5	
$I(0)$, POR (s)	0.0532 (0.0002)	0.1030 (0.0002)
R_g (POR, nm) (s)	7.5 (0.04)	7.7 (0.02)
D_{max} (nm)	26	26
Quality of fit, CorMap P / χ^2	0.7/1.04	0.99/0.99
Porod volume (nm^3)	252	255
Shape classification	extended	extended
**MW and hydrodynamics**		
Calculated MW, from amino acid sequence, kDa	*81.9 (monomer)*	*81.7 (monomer)*
MALS protein MW, kDa	78.5	80.6
MALS glycan MW, kDa	26	19.6
MALS MW, kDa (total)	105	100
MW from SAXS data, kDa	113 (106–127)	94 (89–96)

***Ab initio* modeling**		
Method	*DAMMIN*	
Symmetry	P1	
#models used for averaging	15	12
Normalized spatial discrepancy	0.76	0.77
**Quality-of-fit, χ^2, CorMap *P*	1.06/0.23	1.02/0.65
**Rigid body modeling**		
Method	*AF2/X-ray/CORAL structure*	
Symmetry	P1	
Quality-of-fit, χ^2 /CorMap *P*	1.17/0.072	1.10/0.293
Top model name	PK5su28.pdb	PM5sub6.pdb
**SASBDB accession codes**	SASDPF3	SASDPG3

* The sequence of PTPRK used in this study is isoform 2 in the UniProt entry, possessing an alanine insertion relative to canonical sequence at position 731.

** Of final refined single model derived from the n-model cohort.
